# Publisher Correction: Disposable silicon-based all-in-one micro-qPCR for rapid on-site detection of pathogens

**DOI:** 10.1038/s41467-020-20396-6

**Published:** 2020-12-14

**Authors:** Estefania Nunez-Bajo, Alexander Silva Pinto Collins, Michael Kasimatis, Yasin Cotur, Tarek Asfour, Ugur Tanriverdi, Max Grell, Matti Kaisti, Guglielmo Senesi, Karen Stevenson, Firat Güder

**Affiliations:** 1 Department of Bioengineering, Imperial College London, London, SW7 2AZ UK; 2 Department of Future Technologies, University of Turku, 20500 Turku, Finland; 3 Moredun Research Institute, Pentlands Science Park, Bush Loan, Edinburgh, EH26 0PZ UK

**Keywords:** Sensors and probes, Electrical and electronic engineering, Sensors and biosensors

Correction to: *Nature Communications* 10.1038/s41467-020-19911-6, published online 2 December 2020.

The original version of this Article contained an error in the title, which was previously incorrectly given as ‘Disposable silicon-based all-in-one micro-qPCR for apid on-site detection of pathogens’. The correct version states ‘rapid’ in place of ‘apid’.

This has been corrected in both the PDF and HTML versions of the Article.

