# Supplementary material for: Disposable silicon-based all-in-one micro-qPCR for apid on-site detection of pathogens
Source: Nat Commun. 2020 Dec 2;11:6176. doi: 10.1038/s41467-020-19911-6 (PMC7710731; doi:10.1038/s41467-020-19911-6)
Supplement: Supplementary file 1 — Supplementary Information [file 41467_2020_19911_MOESM1_ESM.pdf]

Supplementary Information

Disposable Silicon-based All-in-One Micro-qPCR for Rapid On-Site Detection of Pathogens

Estefania Nunez-Bajo[1], Alexander Silva Pinto Collins[1†], Michael Kasimatis[1†], Yasin Cotur[1†], Tarek Asfour[1†], Ugur Tanriverdi[1], Max Grell[1], Matti Kaisti[1,2], Guglielmo Senesi[1], Karen Stevenson[3], Firat Güder[1*]

[1]Department of Bioengineering, Imperial College London, London SW7 2AZ, UK

[2]Department of Future Technologies, University of Turku, 20500 Turku, Finland

[3]Moredun Research Institute, Pentlands Science Park, Bush Loan, Edinburgh, Scotland EH26 0PZ, UK

† These authors contributed to the work equally

* Corresponding Author

Dr. F. Güder, e-mail: guder@imperial.ac.uk

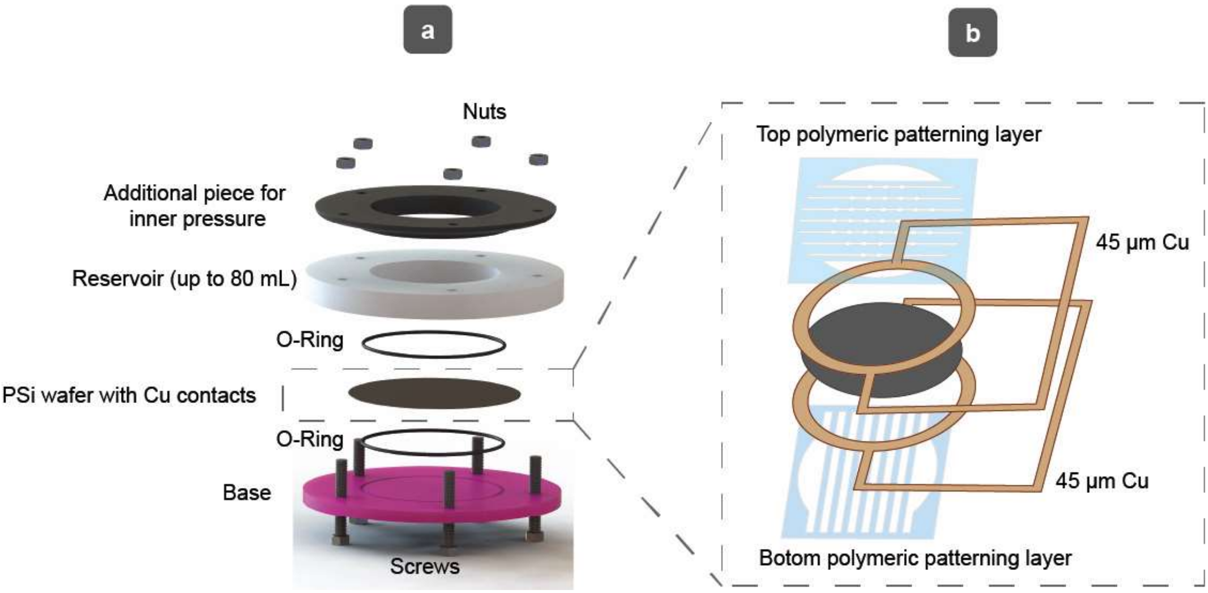

Supplementary Figure 1. Custom holder for electroplating. Schematic illustrations of **a** the holder and **b** integration of thin copper contacts for metal electroplating. To achieve high uniformity when electroplating across the wafer, we have designed, and 3D printed with polylactic acid (PLA) a custom holder which has a circular contact around the edges of the wafer (**Supplementary Figure 1a**). After the incorporation of the polymeric patterning layer on the porous silicon wafer, the surface is not completely flat and the rounded copper contacts (40 µm thicker) must be incorporated, at the top and bottom, between the polymeric layer and the silicon wafer in order to guarantee a good electrical contact for the metal electroplating (**Supplementary Figure 1b**). Then, the reservoir is placed on the top and the holder, closed through five screws and filled with 20 mL of plating solution. Since we observed a progressive leaking of the solution with the time, rubber O-rings were integrated in both, base and reservoir, pieces and a third component was incorporated in order to apply an inner pressure in the region of the O-rings. Once one of the sides of the wafer is electroplated, the plating solution is extracted, and the holder is opened to flip the wafer with contacts. Finally, the holder is closed again through the screws and the plating solution is added again.

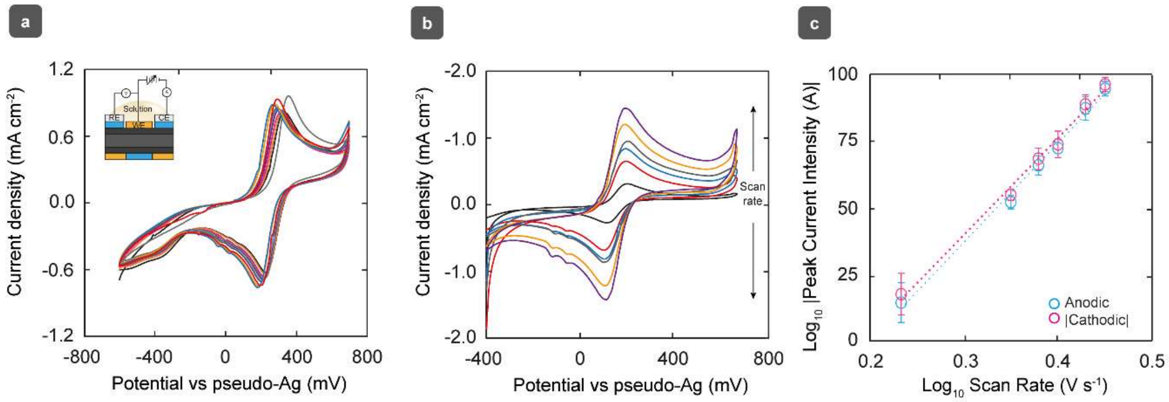

Supplementary Figure 2. Calculation of electrocatalytically active area through Randles-Sevcick. CVs in 2 mM $K_3Fe(CN)_6$ solution (0.1 M KCl) sweeping the potential from -600 to +700 mV **a** at 100 mV s^{-1} for ten consecutive scans and **b** at 10, 50, 75, 100, 150 and 200 mV s^{-1}. **c** logarithmic plot of the peak current (i_p) vs. scan rate, with gradients 0.48 and 0.49. Error bars correspond to the standard deviation from measurements obtained using different Trisilix chips (N=3).[1] As **Supplementary Figure 2a** shows, there is no apparent adsorption on the electrode after 10 consecutive cycles. A reversible process takes place with a difference between anodic and cathodic peak potentials of 87±8.5 mV. The ratios between anodic and cathodic peak current densities (j_{pa}/j_{pc} absolute value) is 0.93±0.04. According to the Randles-Sevcick equation for a flat electrode and for diffusion-controlled processes at 25 °C. [2-5] $\boldsymbol{i_p} = (2.69\cdot10^5)\boldsymbol{n}^{3/2}\ \boldsymbol{A}\ t^{1/2}\ \boldsymbol{C^*}\ \boldsymbol{v^{1/2}}$. Where $\boldsymbol{i_p}$ is the peak current (A), $\boldsymbol{n}$ is the number of electrons transferred (n = 1 for ferrocyanide), $\boldsymbol{A}$ the effective area of the electrode (cm^2), D is the diffusion coefficient of ferrocyanide in aqueous solutions ($7.60x10^{-6}$ cm^2 s^{-1}), $\boldsymbol{C^*}$ is the concentration ($2x10^{-6}$ mol cm^{-3}) and $\boldsymbol{v}$ is the scan rate (V s^{-1}). Cyclic voltammograms, as those shown in Supplementary Figure 2b, were recorded using three different devices without washings between scans. The gradient of the logarithmic plot peak current intensity *vs* scan rate (**Supplementary Figure 2c**) was 0.48±0.03 (R^2 = 0.994) and 0.49±0.03 (R^2 = 0.993), for anodic and cathodic processes, respectively, which corresponds to the value associated with the semi-infinite diffusion of the electroactive species to the electrode in accordance with the Randles-Sevcik equation. Taking this excellent fitting into account, the electroactive area can be calculated from the slope of the linear fitting of i_p vs $\upsilon^{1/2}$ without readjusting the Randles-Sevcik equation[2-5]. Calculated effective areas were 22±0.3

mm^2 and 22±0.4 mm^2 from anodic and cathodic data, respectively. These values are at least the double of the geometric area (9.6 mm^2).

Specification	Value
Resistance (25°C) (ohm)	4.40 ± 0.442 (n=5)
[a]B-constant (25-50°C) (K)	663 ± 52.4 (n=5)
Operating Temperature Range (°C)	0-110
[b]Thermal Time Constant (s)	24.39 ± 1.24 (n=3)
[c]Thermal dissipation constant (mW °C^{-1})	19 ± 1.4 (n=3)

a $B = \ln(R/R_0)/(1/T - 1/T_0)$ where R_0 and T_0 are the resistance and temperature at 25 °C, respectively [1,2]
b Determined by self-heating of the device to 90°C (T_1) and measuring the time taken to fall to 63.2% of the difference between ambient temperature (T_0) and T_1 upon disconnecting the power source.
c Determined by measuring the required power to raise the device temperature 1°C over ambient.

Supplementary Table 1. Specifications of the Si-based thermistor. The thermal characterization of the Si-based thermistor was performed using a Keithley 2450 SourceMeter which allowed the application of different electrical currents for heating and measurement of the voltage through the thermistor at a constant current of 10 mA. Electric measurements were taken on chips housed in a 3D-printed ABS holder connected to the SourceMeter and the temperature was measured using a thermocouple. Experiments were based on industry measures as detailed in typical NTC thermistor datasheets[6,7] .

The degree of precision on the temperature control of the NA amplification chamber is related to the accuracy in establishing the resistance–temperature correlation curve for an Ohmic resistor: $R = R_0(1 + \alpha(T\text{-}T_0))$ where R and R_0 are resistances of the thermistor at temperatures T and T_0 (reference temperature), and α is the temperature coefficient of resistance. This equation is used to convert the resistance reading (calculated from the voltage through the diode connected to the custom board, measured at a constant current of 10 mA every 0.5 s) to the temperature reading of the thermal camera. When R/R_0 is plotted against $T\text{-}T_0$, linear graphs (Figure 3b) with a regression coefficient of 0.9991±0.0007 are obtained using 5 different devices. The slope of this linear graph gives a negative α value of 6.1±0.4 x 10-3 °C^{-1}. With the well-calibrated temperature sensors, fast thermal cycling (heating and cooling gradients of 3.2 and 2.5 °C·s^{-1}, respectively) of the NA amplification chamber (Figure 3e) is achieved with a temperature precision of ±1.3 °C when the values measured by the thermal camera are compared with those of the recorded resistances by the MATLAB-based interface.

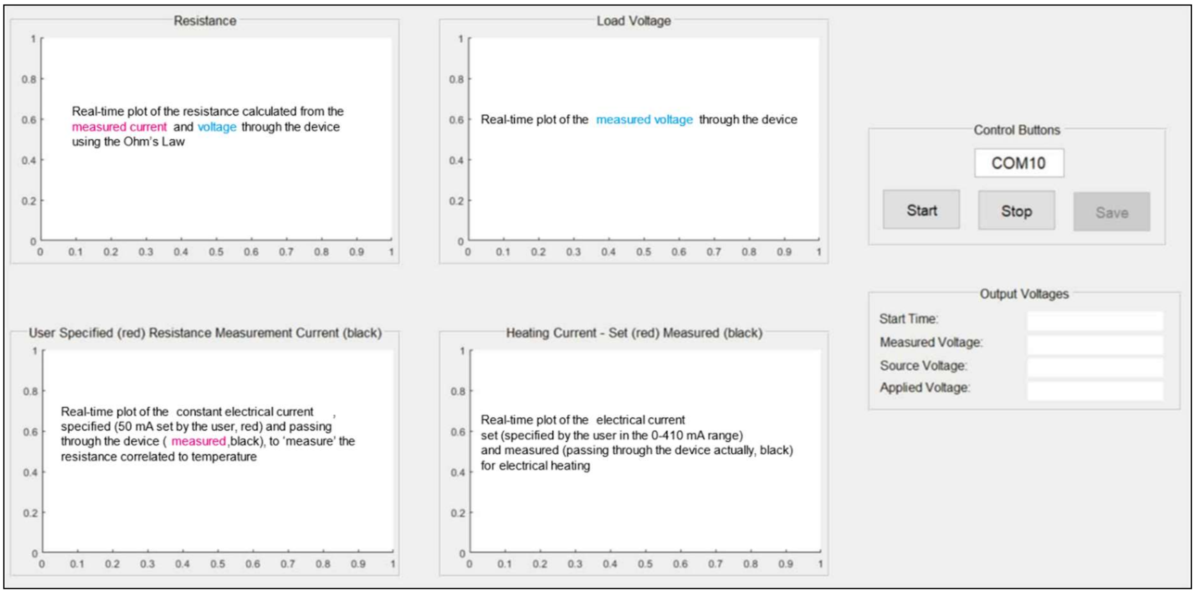

Supplementary Figure 3. MATLAB-based graphical user interface. The X axis of every of the four screens shows the time of the recording in seconds and Y axis shows the resistance in Ohms (upper left screen), the voltage in volts (upper right screen) and the current in amps (bottom screens).

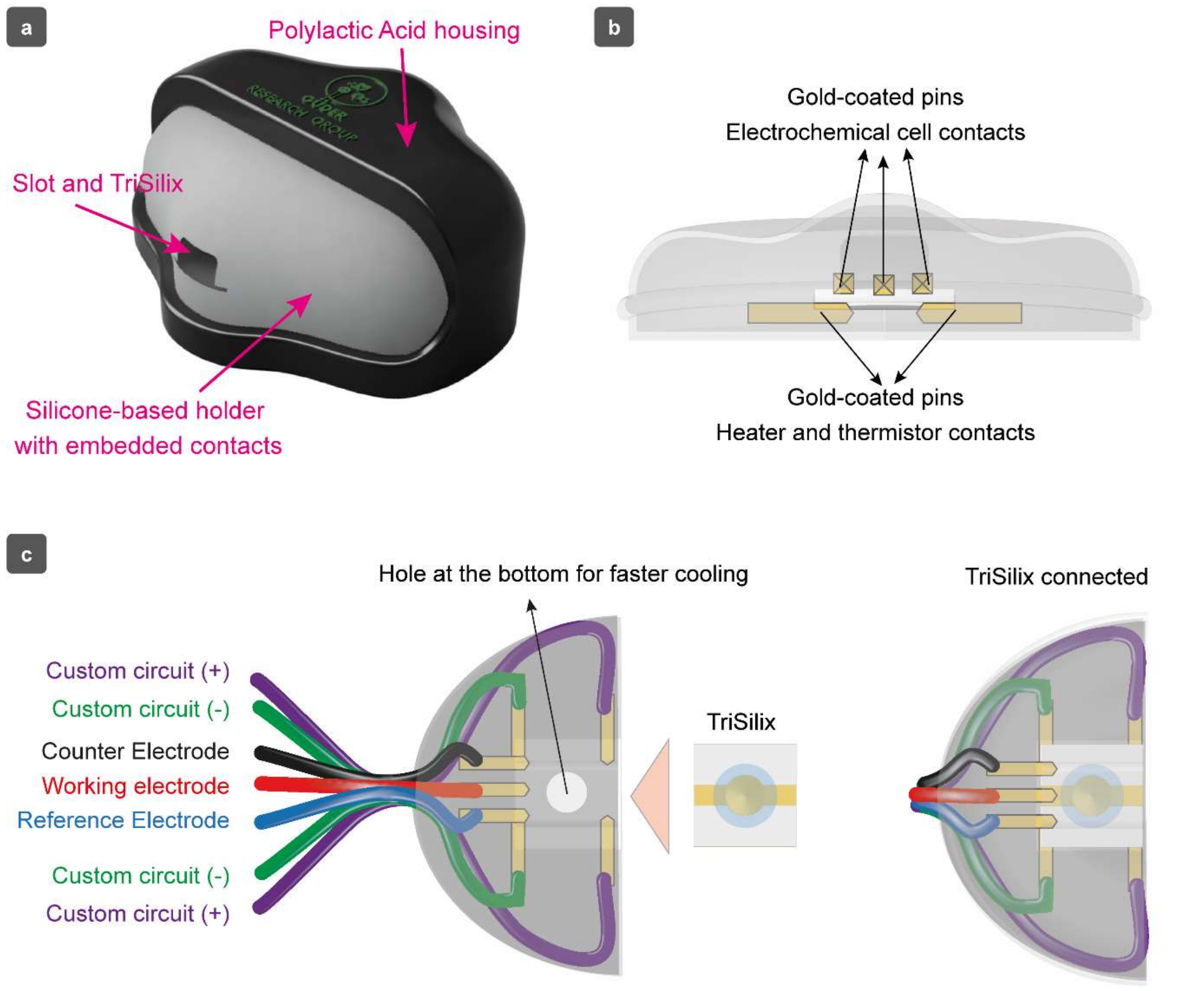

Supplementary Figure 4. Silicone-based sample holder in 3D-printed PLA housing. **a** Front view of the holder with contacts for trimodal operation of Trisilix. Schematic representations of **b** the distribution of embedded contacts and **c** connection of Trisilix (top view) for trimodal operation using the holder.

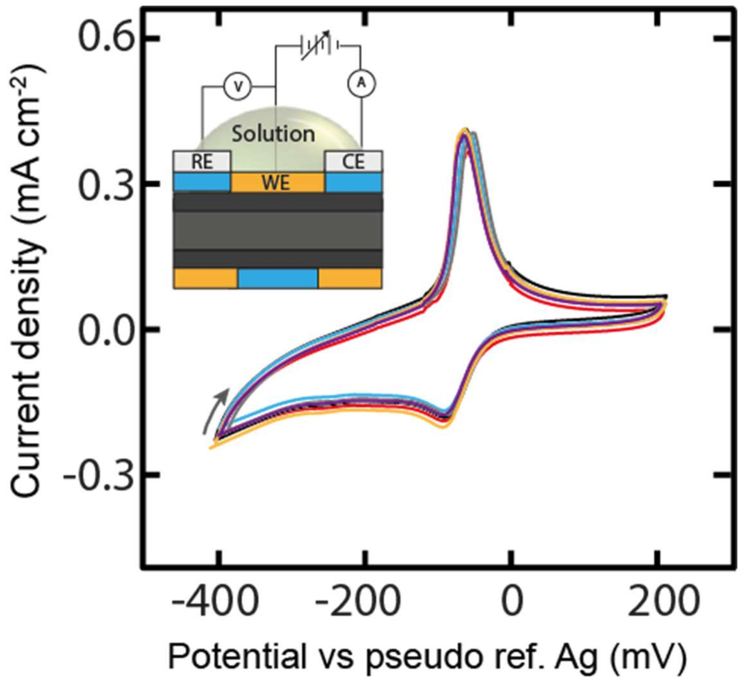

Supplementary Figure 5. Multicyclic voltammetry of Methylene Blue. CVs recorded in 125 μg mL^{-1} MB solution in 10 mM PBS pH 7.0 sweeping the potential ten times (10 scans) from -400 to 200 mV *vs* pseudo reference Ag. Scan rate=100 mV s^{-1}.

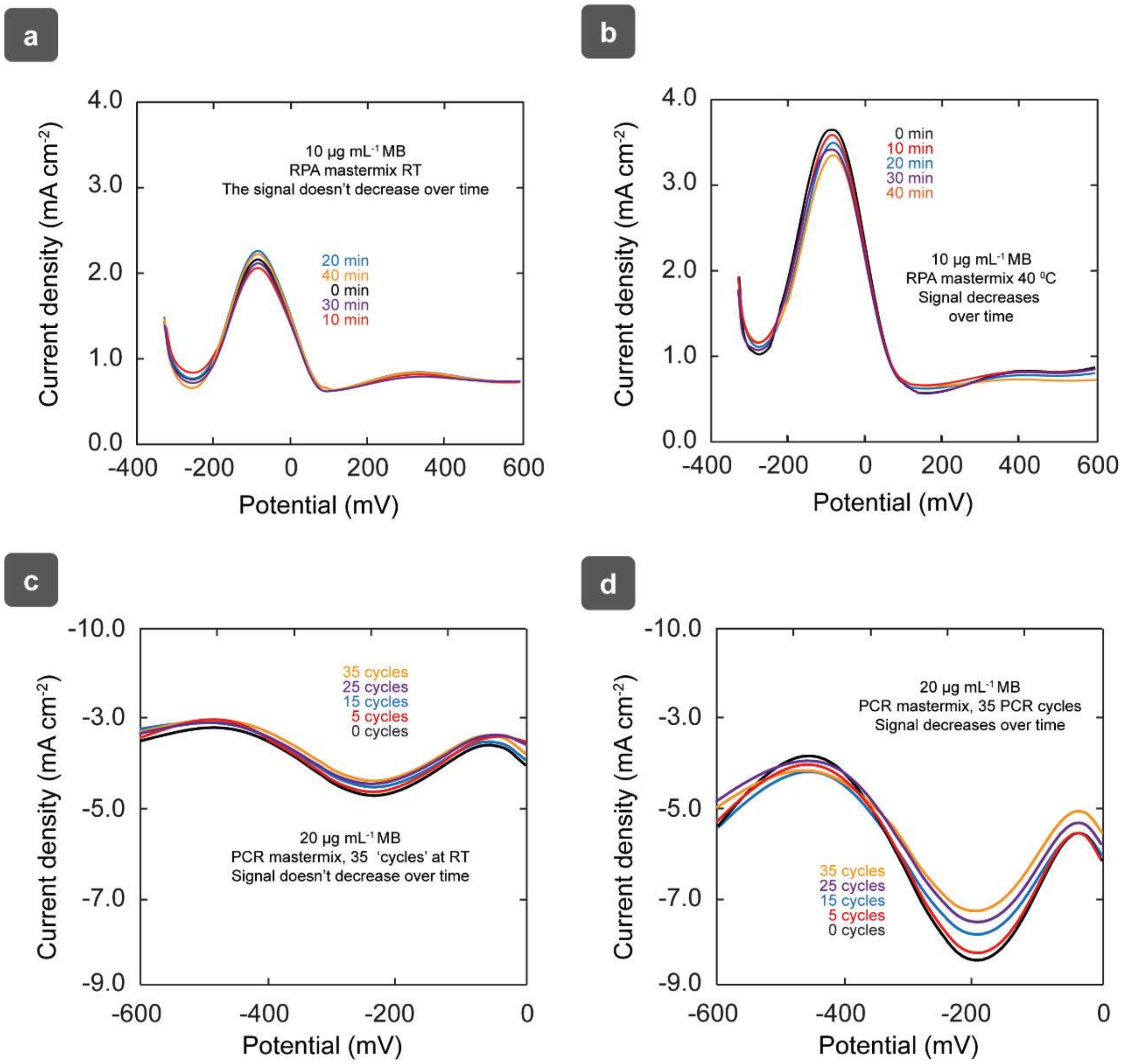

Supplementary Figure 6. Methylene Blue signal stability studies under nucleic acid amplification conditions. Representative SWVs from those recorded every 2.5 min in RPA solutions without DNA (RPA mastermix) under isothermal conditions at **a** room temperature and **b** 40°C during 40 min. Representative SWVs from those recorded every 5 cycles in PCR solutions without DNA (PCR mastermix) **c** at room temperature and **d** under the programmed thermal cycling conditions during 35 cycles. E_{sw}: 100 mV; E_s: 5 mV; *f*:50 Hz.

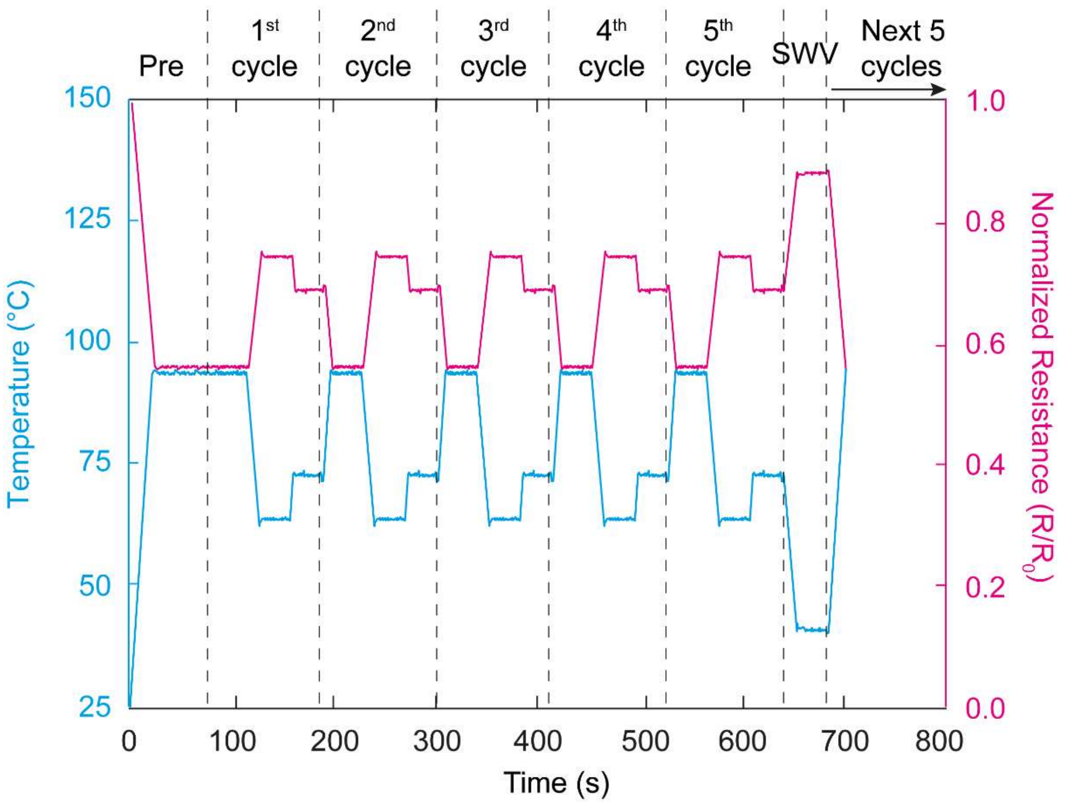

Supplementary Figure 7. Program of temperatures over time during PCR experiments. Thermal cycling curves of the first 5 cycles in qPCR experiments. Dashed lines delimitate each of the 5 PCR cycles performed before SWV measurements. Each cycle of qPCR consisted of three steps: (i) denaturing at 94°C for 30 sec, (ii) annealing at 63°C for 30 sec and (iii) extension 72°C for 30 sec. In addition to these three steps, every fifth cycle, we performed SWV at 40°C (maintained for 30 s) to determine the peak current intensity in relation to the redox processes involving MB. The program of currents to heat the chamber during a PCR cycle consisted of a first step of preheating applying a current of 410 mA for 23 s and 400 mA for 30 s. Then, 5 times the following program: i) denaturalization applying 410 mA for 17 s (except for the first cycle) and 400 mA for 30 s; ii) primer annealing applying 0 mA for 10 s and 270 mA for 30 s; iii) extension applying 315 mA for 3 s and 310 mA for 30 s; iv) electroanalysis applying 0 mA for 12 s and 170 mA for 30 s. Finally, to reach 94 °C for the start of the next series of 5 cycles, a current of 410 mA is applied for 17s.

	0 fg		10 fg		20 fg		50 fg		100 fg	
**Cycle**	$\bar{x}_1$	σ_1	$\bar{x}_2$	σ_2	$\bar{x}_2$	σ_2	$\bar{x}_2$	σ_2	$\bar{x}_2$	σ_2
**20**	0.86731	0.06477	0.82430	0.01407	0.82262	0.05387	0.77760	0.05537	0.69275	0.03785
**25**	0.83955	0.06595	0.79949	0.01548	0.75743	0.04330	0.68505	0.04606	0.54953	0.02193
**30**	0.76571	0.03141	0.73416	0.03095	0.64258	0.04186	0.53888	0.05483	0.34985	0.03988
**35**	0.71632	0.02257	0.69794	0.03856	0.57759	0.04511	0.30661	0.05928	0.14516	0.03616
**t-score 20th cycle**			1.1240		0.9188		1.8233		4.0301	
**t-score 25th cycle**			1.0244		1.8029		3.3269		**7.2277**	
**t-score 30th cycle**			1.2395		4.0749		**6.2177**		**14.1890**	
**t-score 35th cycle**			0.7127		**4.7635**		**11.1869**		**23.2055**	
**t-score 35th cycle (20 fg-50fg difference)**					**6.3005**					

Supplementary Table 2. Student's t-test. Experimental data and calculated t-score values from qPCR experiments performed in 0 fg, 10 fg, 20 fg, 50 fg and 100 fg MAP DNA solutions (n=3). $\bar{x}_1$ and $\bar{x}_2$ are the average values of the normalized signal (electroanalytical signal) for the populations 1 (0fg) and 2 (10, 20, 50 or 100 fg) , respectively, at a given PCR cycle, and σ_1 and σ_2 are their standard deviations. We calculated the t-score using the following equation: $t_{score} = \frac{(\bar{x}_1 - \bar{x}_2)}{\sqrt{\frac{\sigma_1^2}{n_1}+\frac{\sigma_2^2}{n_2}}}$. For a level of significance of α=0.05 (2 tailed) and $d_f = 2$ (N=3, therefore degrees of freedom $d_f = N$-1=2), the critical t-value, t_{crit}=**4.3030**. The application of the t-Student's test at a 0.05 significance level for a range of concentrations from 0 to 100 fg demonstrated that there were no statistically significant differences between the values given by qPCR for DNA solutions containing 10 fg and blank while there were statistically significant differences (data highlighted in the table in bold) for 20, 50 and 100 fg at the 35th, 30th and 25th cycles, respectively. The lowest experimentally detectable concentration was 20 fg. Using the method described by Forootan et al. we also calculated the theoretical limit of detection.[8,9] Working at 95% confidence, we calculated the LOD using the formula $LOD = LOB + 1.645\ \sigma_{20\,fg} = 0.827$. Where σ_{20fg} is the standard deviation of the electroanalytical signal generated by the sample containing 20 fg of DNA (*i.e.* the lowest measurable amount) and LOB the limit of the blank calculated as $LOB = \bar{x}_{Blank} + 1.645\ \sigma_{Blank} = 0.753$. Where $\bar{x}_{Blank}$ and σ_{Blank} are the average and standard deviation of the blank at the 35th cycle. We created a semi-log linear calibration

curve (not shown) using the electroanalytical signals at the 35th cycle for 20 fg, 50 fg and 100 fg MAP DNA which was described as $\frac{j_{35}}{j_0} = (-0.6219)\log_{10} x + (1.3797)\quad [R^2 = 0.9957]$. Where j_0 and j_{35} are the current densities at the 0 and 35th cycles, respectively, and x the amount of genomic Map DNA. Plugging in y=0.827 yields x ~ 8 fg, hence the theoretical LOD is 8 fg.

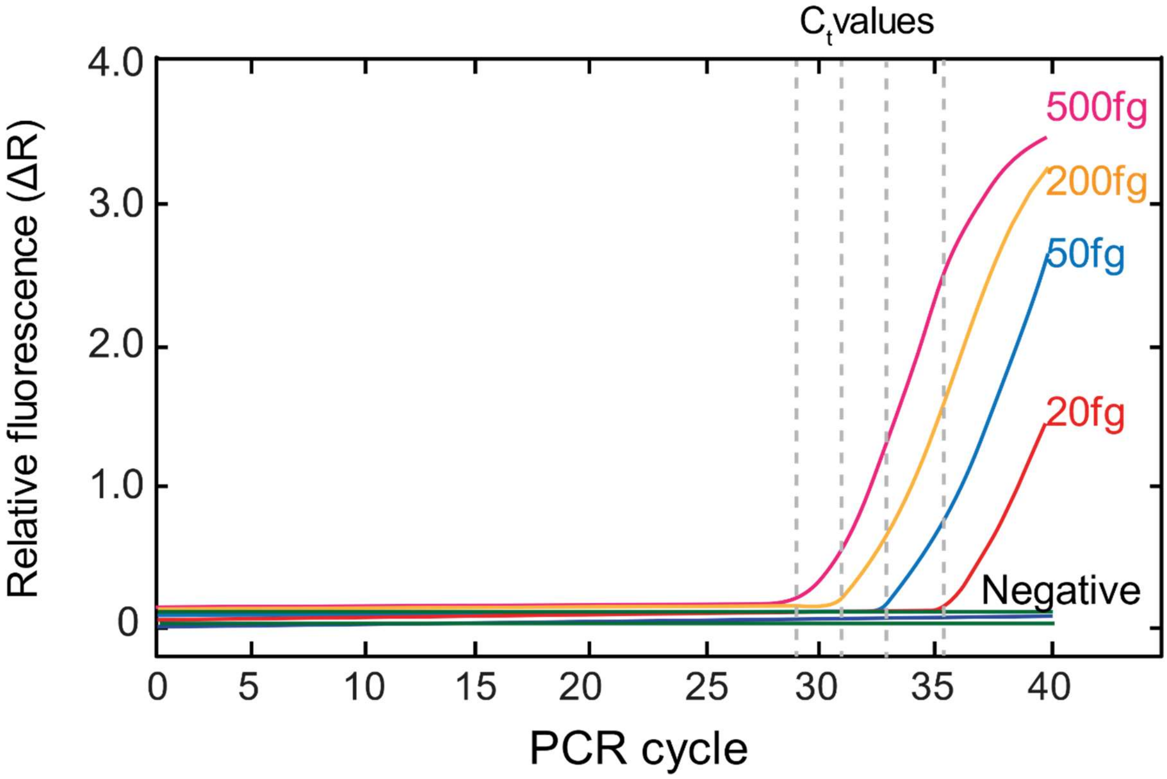

Supplementary Figure 8. qPCR results of MAP K10 DNA using a standard method.

MAP K10 DNA amplification plot using the IDVet commercial qPCR test for Johne's disease. The set cut-off for positives is C_t 40 and samples with C_t below 33 are re-tested as standard to confirm the original positive result.

Product	Conc	Supplier	Ref	Amount	Purchase Price	Spent quantity	Spent Cost	Num. batches	Cost per device
**HF**	50%	Sigma	30107-500ML-M	500	3.6	13.8 mL	0.10	1	0.0027
**H_2O_2**	30%	VWR	8.22287.2500	2500	14.04	13 mL	0.07	1	0.0020
**H_2SO_4**	98%	VWR	20700.420	2500	9.43	30 mL	0.11	1	0.0031
**$KAuCl_4$**	99.995%	Sigma	450235-250MG	0.25	62.19	300 mg	0.75	1	0.0203
**$KAu(CN)_2$**	99.95%	Sigma	379867-250MG	0.25	108.68	116 mg	5.01	3	0.0451
**KCN**		Sigma	60178-25G	25	19	52 mg	0.04	3	0.0004
**AgCN**		Sigma	184535-10G	10	48.68	73 mg	0.36	3	0.0032
**Cu-PET**	20/23 um	UK Insulations Ltd		650 mm 25 m (162500 cm^2)	3.53	5 pieces 256 cm^2	0.03	1	0.0008
**PE**		Kite UK		100 units	2.71	3 units	0.08	1	0.0022
**p-Si wafer**		Inseto		1 unit	9.92	1 unit	9.92	1	0.2681
**TOTAL**									0.35

Supplementary Table 3. Table showing the breakdown of the cost after wafer-scale manufacturing. All prices are in USD.

Supplementary References

1. Bard, A.J, Faulkner, L.R. Electrochemical Methods: Fundamentals and Applications, Willey, New York, 2001.
2. Grewal, Y. S. *et al.* Label-free electrochemical detection of an Entamoeba histolyticaantigen using cell-free yeast-scFv probes. *Chem. Commun.* **49**, 1551–1553 (2013).
3. Shetti, N. P., Nayak, D. S., Malode, S. J. & Kulkarni, R. Electrochemical sensor based upon ruthenium doped TiO2 nanoparticles for the determination of Flufenamic acid. *J. Electrochem. Soc.* **164**, B3036–B3042 (2017).
4. García-Miranda Ferrari, A., Foster, C. W., Kelly, P. J., Brownson, D. A. C. & Banks, C. E. Determination of the electrochemical area of screen-printed electrochemical sensing platforms. *Biosensors* **8**, 53–62 (2018).
5. Zhu, P. & Zhao, Y. Cyclic voltammetry measurements of electroactive surface area of porous nickel: Peak current and peak charge methods and diffusion layer effect. *Mater. Chem. Phys.* **233**, 60–67 (2019).
6. NTC Thermistors *Murata* https://www.murata.com/~/media/webrenewal/support/library/catalog/products/thermistor/ntc/r44e.ashx (2020).
7. NTC Thermistors *Vishay* https://www.vishay.com/docs/29053/ntcappnote.pdf (2017).
8. Shrivastava, A. & Gupta, V. Methods for the determination of Limit of Detection and Limit of Quantitation of the analytical methods. *Chronicles Young Sci.* **2**, 15–21 (2011).
9. Forootan, A. *et al.* Methods to determine limit of detection and limit of quantification in quantitative real-time PCR (qPCR). *Biomol. Detect. Quantif.* **12**, 1–63 (2017).
