# Supplementary material for: Disposable silicon-based all-in-one micro-qPCR for apid on-site detection of pathogens
Source: Nat Commun. 2020 Dec 2;11:6176. doi: 10.1038/s41467-020-19911-6 (PMC7710731; doi:10.1038/s41467-020-19911-6)
Supplement: Supplementary file 2 — Description of Additional Supplementary Files [file 41467_2020_19911_MOESM2_ESM.pdf]

Description of Additional Supplementary Files

File Name: Supplementary Movie 1

Description: Animation demonstrating the components and operation of TriSilix (Tri-modal Silicon-based integrated nucleic acid transducer). The video starts showing the dimensions and components of the chip to perform qPCR: sample reservoir, PCR reagents with redox reporter and silicon-based transducer. Then, it explains the working principles and signaling of the three modes of operation of the silicon-based transducer: i) electrochemical qPCR; ii) electrical heating (Joule heating) and, iii) thermal sensing (thermistor).
